# Laboratory evaluation on the sensitivity and specificity of a novel and rapid detection method for malaria diagnosis based on magneto-optical technology (MOT)

**DOI:** 10.1186/1475-2875-9-207

**Published:** 2010-07-19

**Authors:** Petra F Mens, Raphael J Matelon, Bakri YM Nour, Dave M Newman, Henk DFH Schallig

**Affiliations:** 1 Koninklijk Instituut voor de Tropen (KIT)/Royal Tropical Institute, KIT Biomedical Research, Meibergdreef 39, 1105 AZ Amsterdam, The Netherlands; 2 College of Engineering, Mathematics & Physical Sciences Harrison Building, University of Exeter, Exeter , UK; 3 Blue Nile Institute for Communicable Diseases. University of Gezira, Wad-Medani, Sudan

## Abstract

**Background:**

This study describes the laboratory evaluation of a novel diagnostic platform for malaria. The Magneto Optical Test (MOT) is based on the bio-physical detection of haemozoin in clinical samples. Having an assay time of around one minute, it offers the potential of high throughput screening.

**Methods:**

Blood samples of confirmed malaria patients from different regions of Africa, patients with other diseases and healthy non-endemic controls were used in the present study. The samples were analysed with two reference tests, i.e. an histidine rich protein-2 based rapid diagnostic test (RDT) and a conventional Pan-*Plasmodium *PCR, and the MOT as index test. Data were entered in 2 × 2 tables and analysed for sensitivity and specificity. The agreement between microscopy, RDT and PCR and the MOT assay was determined by calculating Kappa values with a 95% confidence interval.

**Results:**

The observed sensitivity/specificity of the MOT test in comparison with clinical description, RDT or PCR ranged from 77.2 - 78.8% (sensitivity) and from 72.5 - 74.6% (specificity). In general, the agreement between MOT and the other assays is around 0.5 indicating a moderate agreement between the reference and the index test. However, when RDT and PCR are compared to each other, an almost perfect agreement can be observed (k = 0.97) with a sensitivity and specificity of >95%.

**Conclusions:**

Although MOT sensitivity and specificity are currently not yet at a competing level compared to other diagnostic test, such as PCR and RDTs, it has a potential to rapidly screen patients for malaria in endemic as well as non-endemic countries.

## Background

Initiation of malaria treatment largely depends on good, laboratory confirmed diagnosis. However, in many disease endemic countries clinical diagnosis is the only method used to decide whether or not to treat, since laboratory techniques to confirm the clinical suspicion are considered to be too labour-intensive or not sensitive enough [1,2]. In general, screening of blood slides by microscopy is still considered to be the "gold standard". This method is cheap and simple but labour intensive, time consuming and requires well-trained personnel that can differentiate between the different *Plasmodium *species [3]. In recent years, a variety of rapid diagnostic tests (RDTs), detecting circulating *Plasmodium *antigen(s) in the blood of a patient, has been developed for the diagnosis of malaria and are currently rolled out by the World Health Organization (WHO) [4]. These tests are fast, easy to perform and do not require electricity or specific equipment [5-7], but may be limited in sensitivity (detecting only parasitaemia levels above 200 parasites/μl blood) and concerns have arisen about their stability [8]. Alternative platforms to detect malaria are, therefore, still being developed.

**Figure 1:**
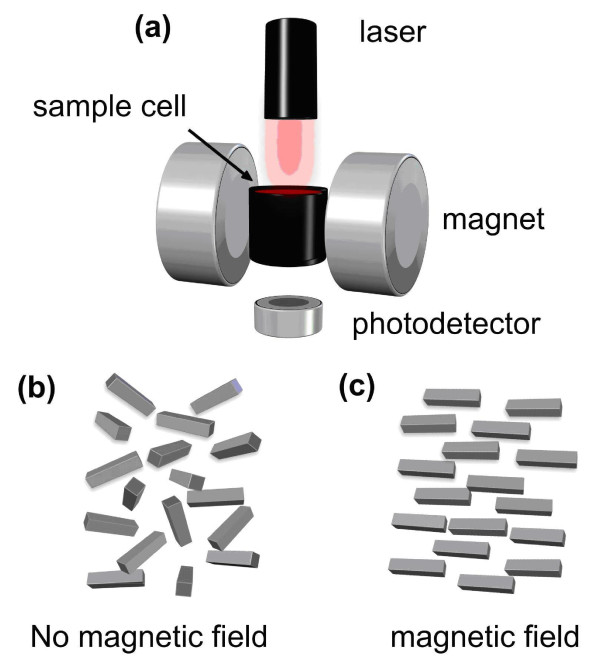
**(a) Schematic diagram of the diagnostic device, a sample cell containing a lysed blood sample is placed within the poles of a magnet supplying a magnetic field up to 0.6 Tesla**. A polarised laser beam passes through the sample and the transmitted intensity is recorded using a photodetector. The intensity is recorded against time with and without a magnetic field. If haemozoin crystals are present, the transmitted intensity is magnetic field dependent. **(b) **When no magnetic field is applied, crystals are randomly orientated and a base line intensity is recorded **(c) **In a magnetic field, the crystals become aligned along the direction of applied field, inducing as an increase in recorded intensity [9].

**Figure 2:**
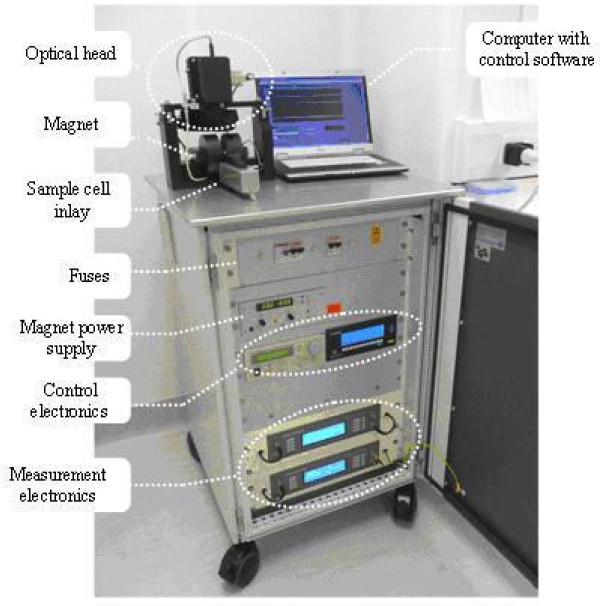
**Overview of the MOT diagnostic device, showing the main components including the optical head, magnet, sample cell inlay and computer controlled unit**.

One such platform for the fast (less than one minute) and easy detection of malaria parasites in a patient blood sample is the Magneto Optical Test (MOT) [9]. Depending on its final sensitivity and specificity, the MOT could potentially be used as a diagnostic device in resource limited settings because of its robustness and the potential of working without electricity (as it can operate on a solar battery) or the need of a cold chain. It would also be suitable for the screening of returning travellers, military personnel returning from a mission or the detection of asymptomatic carriers in a cross sectional or epidemiological survey because of its high throughput nature and limited costs. The MOT principle is based on the following: after invasion of malarial parasites into erythrocytes, *Plasmodium *parasites digest the globin part of haemoglobin. The haem component, which is toxic to the parasite, is converted into haemozoin in the form of rod shaped nano-crystals which, if found in a patient, are indicative of malaria infection. Haemozoin is paramagnetic, i.e. it has a small and positive susceptibility to externally applied magnetic fields under the action of which it develops a small magnetic moment that is not retained when the field is removed [10-12]. When a magnetic field is applied across a potentially infected sample any haemozoin crystals present align within the field whereas in the absence of a field the crystals remain free to orient randomly under the thermal energy of their immediate environment [10-12]. This phenomenon, which is correctly referred to as the Cotton-Mouton effect, can be used to differentiate between a sample containing haemozoin and one without (Figure 1). Laser-based instrumentation able to quantify this phenomenon (Figure 2) has previously shown promising results when attempting the diagnosis of malaria in an experimental setup [9]. To further evaluate the potential of this technology a more elaborate prototype MOT device has been built by one of the partners in the EU sponsored project: "Novel Magneto-Optical Biosensors for Malaria Diagnosis" (the European Commission Framework 6 Programme contract: 016494). This device was extensively evaluated in a laboratory-based trial using a broad spectrum of stored clinical samples and its performance is reported here.

## Methods

### Study population

The study population (Table 1) comprised blood samples (N = 217) obtained from patients visiting various health clinics in disease endemic countries (Sudan, Nigeria, Tanzania, Vietnam) or from returned travellers visiting the outpatient clinic of the Academic Medical Centre (Amsterdam, The Netherlands). All malaria patients were physically examined by experienced medical doctors and a confirmed diagnosis was obtained for each patient by reading of Giemsa-stained slides according to WHO recommendations [13]. Healthy control samples were obtained from 100 blood donations at the Sanquin blood bank in Amsterdam, The Netherlands. By the criteria used to select blood donors (i.e. no physical complaints, no stay in malaria endemic countries for the last five years), it was ensured that these can be considered malaria negative. A small subset of samples with other diseases was included to measure specificity. All patients consented to participate in the study and ethical clearance from the appropriate authorities was obtained when necessary.

**Table 1:** Overview of samples included in the evaluation of the MOT device.

Clinical description	Country of Origin	Number used for analysis
Healthy controls	The Netherlands	100

Microscopically confirmed malaria cases	Sudan	72

Microscopically confirmed malaria cases	Vietnam	8

Sickle cell anaemia patients*	Sudan	20

Sickle cell anaemia patients	Nigeria	5

Sickle cell anaemia patient	Tanzania	1

Arthritis patients	Tanzania	2

B-Thallesemia patients	Tanzania	2

Visceral leishmaniasis patients (returning travels)	The Netherlands	7

Total		217

* This set of samples was not analysed with microscopy, however RDT and PCR testing gave evidence that 3 patients in this group were in fact also suffering from a malaria infection.

### Sample collection and handling

From all patients and controls, 2 ml of venous EDTA blood was collected for DNA extraction and PCR analysis, immunochromatic histidine rich protein-2 (HRP-2) detection and MOT analysis. After collection the blood was stored at -20°C until further use. All blood samples were examined for the presence of *Plasmodium *DNA by conventional PCR detecting all four human plasmodium species, *Plasmodium falciparum, Plasmodium vivax, Plasmodium ovale *and *Plasmodium malariae *as described in previous publications and with a human household gene GAPDH to control for isolation and amplification [14]. If no internal control appeared after amplification the PCR reaction was scored as invalid. The presence of HRP-2 indicating an active or recently passed infection with malaria was detected in 5 μl blood by an immunochromatic assay (RDT) (Paracheck, Orchid Biomedical Systems, Verna, Goa, India), according to the manufacturer's instructions and read after 15 minutes by two independent scientists. Both the RDT and PCR reactions were performed independently by experienced laboratory technicians familiar with the respective tests. Data was scored on a data sheet and communicated to the data manager who entered the data into the database.

### MOT analysis

**Figure 3:**
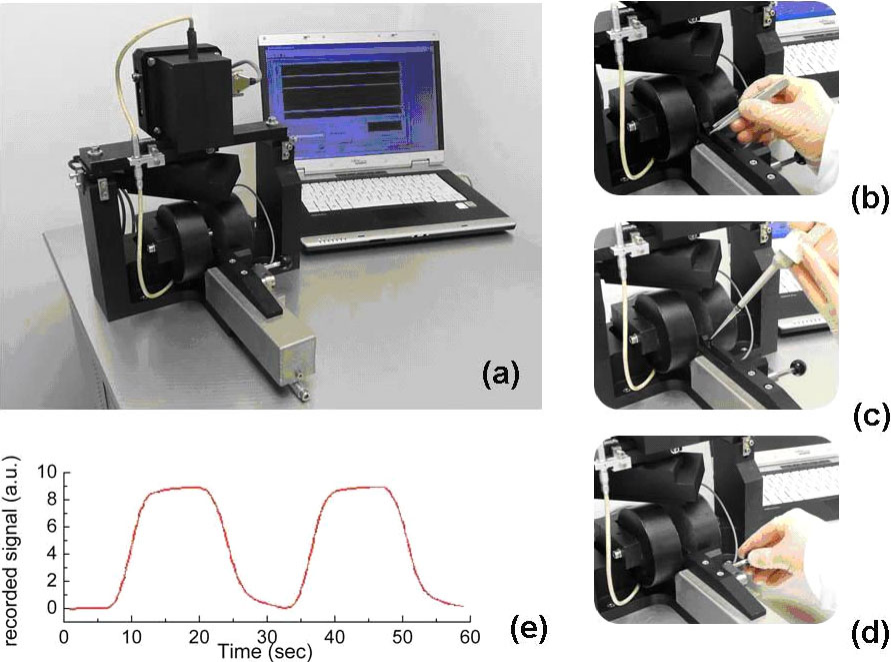
**Photographs of the measurement platform (a) and measurement procedure (b-d)**. **(b) **The operator loads an empty sample cell within the device's loading mechanism, **(c) **a lysed blood sample (150 μl) is pipetted into the sample cell, **(d) **the sample cell is placed within the magnet using the sample loading mechanism. Once the loading procedure is complete an automated measurement routine can be initiated via the dedicated control software. Typical data output is shown in **(e)**, recorded from a 32,500 parasites/μl sample and clearly showing an intensity change; regions of maximum signal coincide with an applied magnetic field while regions of minimum signal are recorded with no applied field.

All analytical MOT measurements were taken using an experimental instrument having the same specifications and operational methodology described previously (figure 3) [9]. Prior to analysis, the samples were thawed and 100 μl transferred to an Eppendorf tube together with 400 μl cell lysis buffer (pH 7.5 Tris/HCl 50 mM, NaCl 150 mM, Nonidet P40 1%, Sodium deoxycholate 0.5%) before sonicating for 10 seconds. After processing in this manner samples were immediately passed to the MOT instrument operator, an experienced physicist blinded from the available clinical and diagnostic information, who transferred 150 μl of each to one of the glass bottomed sample cells used by the instrument. The cells were then introduced into the instrument and the measurement sequence for the Cotton-Mouton effect initiated. The magnitude of the Cotton-Mouton effect is proportional to the level of haemozoin present in the blood sample. A value measured above the noise floor of the instrument is positively indicative of malaria. The results of MOT testing (i.e. positive or negative) were communicated to a data manager who entered the information into the database.

Calibration of the MOT instrument was by a serial dilution of β-haematin in phosphate buffered saline (PBS) prior to the initial testing of the patient samples. The instrument is sensitive to the presence of haemozoin in the blood down to concentrations of 5 ng/μl or better which as discussed in [9] is considered to be equivalent to between 50 and 100 parasites/μl

### **Statistics**

Before final analysis of the data, samples for which insufficient clinical or analytical information was available were removed from the study. Microscopy performed at initial diagnosis is considered as the golden standard for malaria diagnosis. Results of the MOT assay are consequently tested against this standard and also compared to the RDT and PCR obtained data.

Data were entered in 2 × 2 tables and analysed for sensitivity (i.e. the probability that the assay will be positive when the infection is present) and specificity (i.e. the probability that the assay will be negative when the infection is absent) using the formulas:

Sensitivity=TP/(TP+FN)×100% and Specificity=TN/(TN+FP)×100%

In the equations above, TN represents true negative, TP true positive, FN false negative and FP false positive. In addition likelihood ratios (LR) were calculated. The agreement between microscopy, RDT and PCR and the MOT assay was determined by calculating Kappa values with a 95% confidence interval [15] using Epi-info version 6. Kappa values express the agreement beyond chance and a kappa value of 0.21-0.60 is a moderate, a kappa value of 0.61-0.80 a good and kappa > 0.80 an almost perfect agreement beyond chance.

## Results

In this retrospective laboratory-based evaluation, a total of 217 samples were analysed with three reference tests and the MOT as index test. Samples were collected in 2008 and analysed with MOT in July 2009. In total, 84 samples out of the 217 were confirmed (either microscopy or, in the case of the sickle cell patients, RDT and PCR positive) malaria positive. Parasitaemia of the positive samples ranged from 600 parasites/μl to 85,500 parasites/μl. When these samples were tested with MOT, 65 were scored positive (see Table 2). Seven of the samples analysed with MOT were inconclusive. However, in all the comparisons, the inconclusive results were regarded as negative. Omitting the inconclusive samples from the analysis did not give a significant difference. Of the 133 confirmed negatives, 100 were found negative with MOT giving a sensitivity of 78.3% and a specificity of 74.4%.

**Table 2:** Results of confirmed malaria samples in comparison to the MOT results.

		Confirmed Malaria		
		**Positive**	**Negative**	**Total**

	Positive	65	33	98
	
**MOT**	Negative	19	100	119
	
	Total	84	133	217

Also a direct comparison between the different alternative diagnostic methods (RDT and PCR) and MOT was done. When RDT results were compared to MOT, a sensitivity of 77.2% and a specificity of 72.5% were observed for the MOT test. For PCR, one sample could not be analysed, because the internal control was not amplified, leaving 216 samples for further analysis. If the inconclusive MOT results are not taken into consideration, then a sensitivity of 78.8% was found and a specificity of 74.6% for the MOT test.

Of the 30 samples from endemic negative controls, seven samples were found false positive with the MOT test while being negative with all other employed tests; they originated from Sudanese sickle cell patients. The Sudanese malaria confirmed patients that were missed in the MOT test (n = 17) had a parasitaemia ranging from 5,680 to 78,000 parasites/μl (mean: 32,615 parasites/μl). One sample from Vietnam missed with the MOT analysis had a parasitaemia of 10,000 parasites/μl. One sickle cell patient that was found positive with RDT and PCR for malaria was scored inconclusive with MOT.

In general with all comparisons, the agreement between MOT and the other assays was around 0.5, indicating a moderate agreement between the reference and the index test. However, when RDT and PCR were compared to each other only three samples out of the 216 samples that could be compared were discordant (two PCR negative RDT positive and one vice versa). Of the other samples 135 were negative by both tests and 78 positive by both tests thus showing an almost perfect agreement (k = 0.97), with a sensitivity and specificity of over 95% indicating that the reference tests are performing at a competing level. For a complete overview and statistical analysis of the different comparisons see Table 2, Table 3, Table 4 and Table 5.

**Table 3:** Overview of the RDT results in comparison to the MOT results

		RDT		
		**Positive**	**Negative**	**Total**

	Positive	61	36	97
	
**MOT**	Negative	19	101	120
	
	Total	80	137	217

**Table 4:** Overview of the PCR results in comparison to the MOT results

		PCR		
		**Positive**	**Negative**	**Total**

	Positive	63	34	97
	
**MOT**	Negative	19	100	119
	
	Total	82	134	216

**Table 5:** Statistical analyses of the different comparisons excluding inconclusive PCR or MOT results

comparison	Sensitivity in % (95% CI)	Specificity in % (95% CI)	+ Likelihood ratio (95% CI)	- Likelihood ratio (95% CI)	Agreement %	k-value
Confirmed vs MOT	78.3 (68.3-85.8)	74.4 (65.8 -80.9)	3.0 (2.2-4.1)	0.2 (0.2-0.5)	75.7	0.51

RDT vs MOT	77.2 (66.8-85.1)	72.5 (64.3-79.4)	2.8 (2.1-3.8)	0.3 (0.2-0.5)	74.3	0.48

PCR vs MOT	78.8 (66.6-84.6)	74.6 (66.6-81.2)	3.0 (2.2-4.1)	0.3 (0.2-0.5)	75.1	0.49

PCR vs RDT	98.7 (93.2-99.8)	98.5 (94.8-99.6)	67.6 (17.1-267.8)	0.03 (0.02-0.09)	98.6	0.97

## Discussion

This paper describes an extensive laboratory evaluation of a new and very rapid technology for the diagnosis of malaria by detecting the presence of haemozoin in the blood sample of suspected patients. Although several similar routes for the diagnosis of malaria via detection of the malarial pigment in a patient's blood have been explored in recent years this present technique uniquely utilises the magneto-optical properties of the blood-haemozoin system.

Automated blood count machines, such as Cell-Dyn^® ^(Abbot, Santa Clara, California) utilize flow cytometry techniques to detect haemozoin-containing monocytes (PCM) during routine full blood count (FBC) in research settings but have not been applied in medical practice [16-18]. Such technology performs cell-by-cell analysis of the optical scattering properties of circulating cell suspension yielding information on cell size, internal structure, granularity and surface morphology. FBC results are analysed by visual inspection of granularity/lobularity plot on the instrument's display monitor with certain recorded scattering events considered to represent an HZ containing monocyte. In small trials [16-18], samples from endemic countries suffered from persisting haemozoin-containing white blood cells that resulted in false positive observations and, therefore, the true sensitivity and specificity of these automated methods still have to be confirmed. Significant adjustments to the current software and measurement algorithm of these apparatus have to be implemented to ensure user-friendliness and flag suspicious samples more clearly [18] while extending the measurement to a greater number of cells [19]. However, it is deemed unlikely [20] that the cost (~$40,000), complexity and physical size of commercial flow cytometers will ever decrease sufficiently to get such apparatus to the resource-poor areas most affected by malaria where it is needed. In contrast, the MOT test accesses the total haemozoin load from a given blood sample, including PCM and PRBC, by performing a volumetric test using an apparatus whose relatively simpler measurement principle lends itself to the production of a rapid portable, battery operated point-of-care device at costs one or two order of magnitude lower than currently available Cell-Dyn^® ^apparatus.

The MOT test described has been extensively tested in the present study on a large sample set of positive and negative samples and the results were compared against microscopy, RDTs detecting HRP-2 antigen and PCR. The performance, in terms of sensitivity and specificity, of RDT and PCR employed in the present evaluation was good since there was an almost perfect agreement between the comparative tests. The observed sensitivity/specificity of the MOT test in comparison with clinical description, RDT or PCR ranged from 72.5 - 74.6% (specificity) and from 77.2 - 78.8% (sensitivity) with a very low positive likelihood ratio between 2.8 and 3.0. Although in other studies false positivity is often attributed to circulating antigen from cleared parasites, this can in the present study only explain seven of the false positive samples. All these samples were obtained form Sudanese sickle cell patients that were negative with the other employed tests. It remains unclear whether the false positivity is caused by circulating antigen or is influenced by their sickle cell trait and this should be studied further on a larger set of samples of malaria negative endemic controls. The other false positive samples were obtained from healthy non-endemic controls. These non-endemic samples were obtained from healthy donors of the Dutch blood bank who have not traveled to a malaria endemic country within the last five years before their donation. The false positivity of this specific group of samples is concerning but is possibly attributable to contaminant structures introduced during the hand assembly of the sample cells which is currently conducted without quality control. Cells are fabricated from two components a thin glass window (7 mm in diameter) and a carbon injection moulded cylinder. The single use disposable cell is formed by using pre-cut adhesive inserts to cement the glass window to the cylinder. Although all sample cells were only used once and no contaminants form other samples could explain the false positivity, other contaminants introduced during assembly process, for example particles from the environment, which are free and able to respond to the application of a magnetic field will generate a false signal. Problems associated with sample cells can ultimately be easily addressed by employing an automated assembly and quality control procedure whilst further study on the response of various lysis buffers to magnetic field will fully characterize and possibly reduce their magneto-optical contribution such that changes to the measurement algorithm may be implemented to significantly reduce false positivity.

A substantial number (n = 19) of malaria confirmed cases are being missed by the MOT test. These samples all had a parasitaemia above 1,000 parasites/μl and should be readily detectable by the MOT instrument, which has an estimated analytical sensitivity of between 50 and 100 parasites/μl. There are several possible explanations for these false negatives; the most obvious being associated with the fact that the MOT diagnostic process was developed and calibrated using fresh blood samples spiked with β-haematin or live cultured parasitized red blood cells. The samples used in the present study had been stored at -20°C before testing and some had been freeze thawed several times. This may have caused agglutination of haemozoin crystals resulting in a non-homogenous sample and thus not representative anymore for the original sample. Furthermore, if the agglutinated crystals are not fully sonicated the mobility of the haemozoin under action of the magnetic field will be impaired. Either or both these effects could result in the Cotton-Mouton signal massively under representing the mass of haemozoin present and thus might result in a false negative signal. A second factor that currently further complicates the relationship between parasitaemia and haemozoin and which might also lead to false positivity or false negativity is the wide variation of haematocrit levels between patients. These can result in patients sampled at the same point in the parasites life cycle and found to have identical levels of parasitaemia registering vastly different levels of haemozoin. Variations in haematocrit also impact adversely on the measurement procedure by producing corresponding variations in sample transmittance and hence in the signal level and dynamic range recorded at the optical detector. None of the above issues are currently allowed for by the decision-making algorithms of the MOT instrumentation. Attention is drawn however to the point that although presented as a screening device, with a positive or negative diagnostic output, the MOT technique in returning a magneto-optic signal proportional to the haemozoin concentration (as evidenced by calibration data) together with an optical signal proportional to the haematocrit, offers a means of studying the complex inter-relationship between haemozoin, parasitaemia and haematocrit in blood samples. This may allow correlating haemozoin concentration against parasitaemia for the widest range of haematocrit levels experienced in practice. If necessary this process can be further extended to include the impact of other disease states such as anaemia on diagnostic sensitivity. These clinical parameters could be programmed into the decision-making algorithms of future MOT instrumentation and may substantially improve the performance of the instrument with respect to false reporting.

A commercially viable product must be able to compete with RDT and microscopy and the current sensitivity/specificity and predictive value of MOT is not yet at an appropriate level.

The principle limitations of the current study are the lack of fresh samples, lack of information on haemozoin and haemoglobin levels that may influence the outcome of the result, the limited amount of endemic controls and the influence of hand assembly of the sample cells making it difficult to asses the true potential of the device. These issues can however easily be addressed in a future trail. Having a large range of samples from the same setting with known malaria prevalence could give a better indication on its positive and negative predictive value which is not possible with the current sample set.

It is however very promising that the device has been designed and assembled in less than 3 years after the initial idea with still potential for substantial improvement in the design of the hard- and software. The simplicity of the device, low costs (a sample cell will only cost 25 euro cents), the straightforward operation with a limited number of handling steps and the possibility to operate the instrument without a constant supply of network electricity (i.e. it can operate on a battery) combined with the short assay time of 1 minute creates a technology with great potential for simple malaria screening in malaria endemic as well as non endemic countries.

## Conclusion

This paper describes the evaluation of a novel and very rapid diagnostic device based Magneto Optical Technology (MOT) for the diagnosis of malaria by detecting haemozoin in a small patient blood sample. The MOT technology has been evaluated on a large panel of stored blood samples. Although the sensitivity and specificity are not yet at a competing level compared to other diagnostic test, such as microscopy and RDTs, it has a potential to rapidly screen patients for malaria in endemic as well as non-endemic countries. Therefore, the technique should be evaluated on a panel of fresh blood samples after the necessary adaptations of the device's algorithm.

## Competing interests

The authors declare that they have no competing interests.

## Authors' contributions

PM designed the study, conducted PCR analysis, was involved in sample collection, performed data analysis and wrote the manuscript; RM participated in study design, assisted in designing and building the prototype, performed MOT tests and helped to draft the manuscript; BN supervised sample collection and performed microscopical analysis of clinical samples, commenting on the manuscript; HS study design, RDT analysis, mediated in sample collection and performed statistical analysis and helped in drafting the manuscript; DN conceived the MOT principle, supervision of MOT prototype design and production, writing of manuscript. All authors read and approved the final manuscript.
